# Supplementary material for: Evolution of Status of Trace Elements and Metallothioneins in Patients with COVID-19: Relationship with Clinical, Biochemical, and Inflammatory Parameters
Source: Metabolites. 2023 Aug 9;13(8):931. doi: 10.3390/metabo13080931 (PMC10456601; doi:10.3390/metabo13080931)
Supplement: Supplementary file 1 [file metabolites-13-00931-s001.zip › metabolites-2505655-supplementary.pdf]

Table S1. Clinical parameters of critically ill patients with COVID-19 on the 1st and 3rd days of ICU stay.

n = 86	References values	1st day (Mean (SD))	3rd day (Mean (SD))	Mean differences	P-Value
**Clinical**					
Age (years)	–	62.2 (13.5)		–	–
Exitus	–			–	–
No/*n*		59/86		–	–
Yes/*n*		27/86		–	–
ICU stay (days)	–	20.5 (19.4)		–	–
APACHE II score	–	13.7 (7.7)		–	–
SOFA score	–	4.7 (2.5)	5.0 (3.1)	+0.3	0.190
MV (days)	–	15.0 (18.6)	15.0 (18.6)	–	–
MAP (mmHg)	70.0–105.0	95.5 (13.8)	93.2 (10.4)	–2.3	0.560
HR (bpm)	60.0–100.0	76.8 (21.0)	69.6 (18.6)	–7.2	0.142
BR (brpm)	15.0–20.0	26.8 (6.8)	22.1 (6.1)	–4.7	**0.001**
FiO_2 (%)	>0.68	0.8 (0.2)	0.6 (0.2)	–0.2	**0.001**
PaO_2/FiO_2	200.0-300.0	183.4 (111.7)	207.9 (84.5)	+24.5	0.272
PEEP (cm H_2O)	2.0–5.0	12.9 (2.3)	12.0 (2.1)	–0.9	**0.038**

n = 86. Data expressed as mean (standard deviation) unless otherwise stated. Paired Student's t-test was used to compare the evolution of the variables between the 1st and 3rd days of ICU stay. Mean differences represent the difference between the value of the 3rd day and the value of the 1st day of ICU stay. Significant P-Values are represented in bold. Statistical significance = $P<0.05$. Abbreviations: APACHE-II = Acute Physiology and Chronic Health Evaluation-II; BR = Breathing Rate; bpm = beats per minute; brpm = breaths per minute; HR = Heart Rate; MAP = Mean Arterial Pressure; MV = Mechanical Ventilation; PaO_2/FiO_2 = Partial Oxygen arterial pressure/Fraction of Inspired Oxygen; PEEP = Positive End-Expiratory Pressure; SD = Standard Deviation; SOFA = Sequential Organ Failure Assessment.

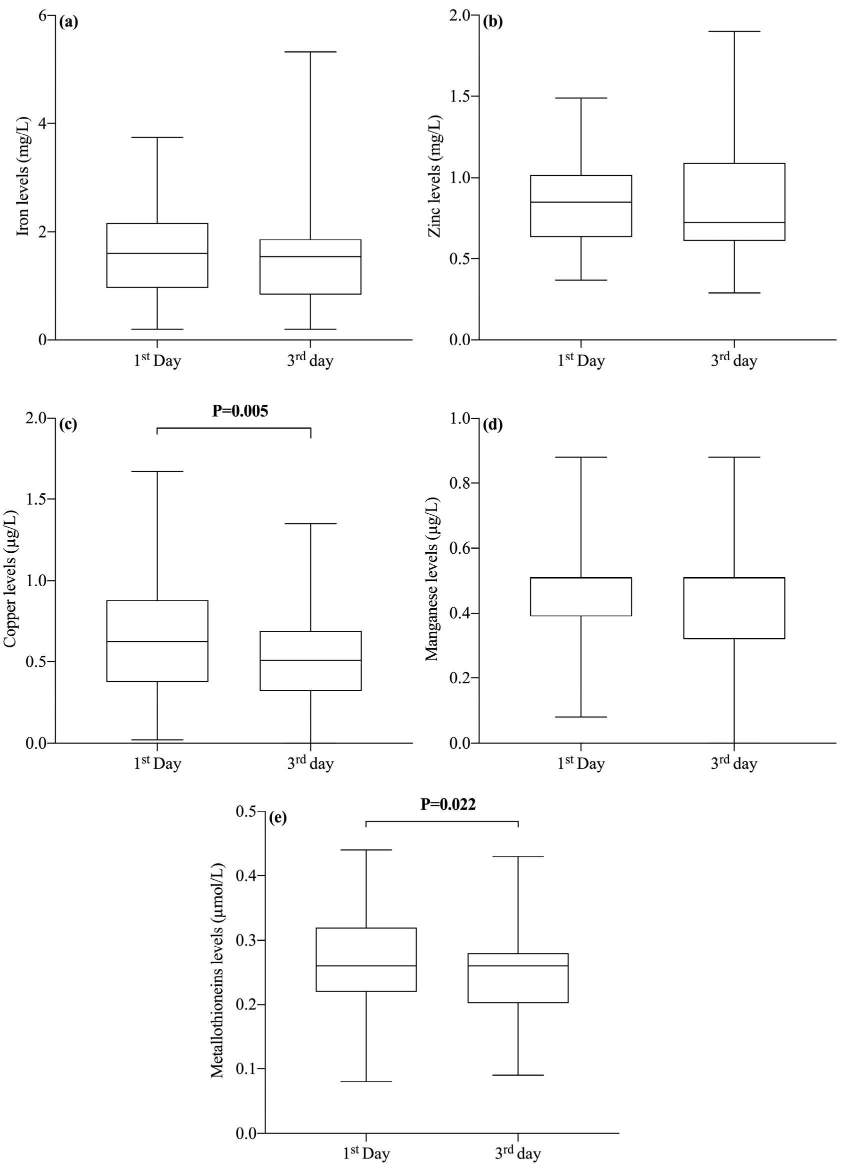

Figure S1. Mineral, and metallothionein values of critically ill patients with COVID-19 on the 1st and 3rd days of admission to the ICU. *n* = 86. Data expressed as mean (standard deviation). Paired Student's t-test was used to compare the evolution of the variables between 1st and 3rd days of the ICU stay. Significant P-Values are represented in bold. Statistical significance = $P<0.05$.
